# Dyslipidemia, hormonal profiles, and sleep characteristics in men with androgenetic alopecia: an analysis based on data from the EPISONO study

**DOI:** 10.1007/s42000-025-00751-2

**Published:** 2026-05-04

**Authors:** Ellen M. S. Xerfan, Maingredy Rodrigues Souza, Paula Araujo, Sergio Tufik, Monica L. Andersen

**Affiliations:** 1 Instituto do Sono, Associação Fundo de Incentivo à Pesquisa (AFIP), São Paulo, Brazil; 2 Departamento de Psicobiologia, Universidade Federal de São Paulo, Rua Napoleão de Barros, 925 Vila Clementino, São Paulo/SP, 04024-002 Brazil; 3 Departamento de Biorregulação, Instituto de Ciências da Saúde, Universidade Federal da Bahia, Salvador, Bahia Brazil

**Keywords:** Sleep, Obstructive sleep apnea, Androgenetic alopecia, Triglycerides, Dyslipidemia

## Abstract

**Background:**

Androgenetic alopecia (AGA), the most common form of male hair loss, is primarily driven by genetic predisposition and androgen activity. Emerging evidence suggests that AGA is associated with metabolic dysfunction and sleep disorders, but their contribution to its pathogenesis remains unclear.

**Aims:**

To examine the hormonal, metabolic, and sleep profiles of adult men with self-reported AGA and their potential relevance as risk factors for this condition.

**Methods:**

This cross-sectional study used data from the 4th Edition of the São Paulo Epidemiological Sleep Study (EPISONO 2018). Participants self-reported AGA, completed sleep questionnaires, underwent polysomnography, and provided blood samples for laboratory measures. A first analysis of all included men was performed, followed by a propensity score matching (PSM), which was applied to match AGA and non-AGA groups by age, body mass index, waist-to-hip ratio, diabetes, and metabolic syndrome.

**Results:**

Of the 769 EPISONO participants, 317 were men and 307 answered the AGA question with 64 reporting AGA and 243 classified as non-AGA. Before PSM, the AGA group was older (p = 0.010) and presented higher triglycerides (p = 0.015), lower HDL cholesterol (p = 0.041), lower total (p = 0.025) and free testosterone (p = 0.011), and higher prevalence of diabetes (p = 0.043), metabolic syndrome (p = 0.012), and moderate-to-severe obstructive sleep apnea (OSA; p = 0.041). Subjective sleep measures did not differ between groups. After PSM, 64 matched pairs were analyzed. All anthropometric, hormonal, metabolic, and sleep measures became comparable between groups and the association with OSA no longer persisted. Across univariate and multivariable logistic regression models, before and after PSM, triglycerides remained the only factor associated with AGA (p = 0.039).

**Conclusions:**

This study reinforces the link between AGA and lipid metabolism, with elevated triglycerides as a predictor, highlighting the importance of metabolic screening in men with AGA while supporting further investigation of lipid-related mechanisms.

## Introduction

Androgenetic alopecia (AGA) is the most common cause of non-scarring hair loss in adults of both sexes, although it is more prevalent in men [1]. Its occurrence rises with age, from 30% at 30 years old to 80% by age 70 [2, 3]. The underlying mechanism involves an excessive response to androgens, primarily influenced by genetic factors that increase susceptibility to progressive hair loss. Overactivation of androgen receptors appears to modify the transcriptional pattern of dermal papilla cells, disrupting hair cycle dynamics, shortening the follicular growth phase (anagen), and ultimately leading to follicular miniaturization [4–6].

Although the classical view is that the etiology of AGA primarily stems from androgen metabolism linked to genetic predisposition, recent evidence suggests that its pathophysiology is also associated with local inflammation [7, 8]. Androgens appear to induce the expression of inflammatory mediators in dermal papilla cells, and this microinflammation is thought to contribute to follicular apoptosis and subsequent miniaturization [9]. In this context, it is reasonable to consider that chronic and systemic inflammatory conditions, such as diabetes, dyslipidemia, hypertension, and obstructive sleep apnea (OSA) [10–13], could exacerbate microinflammation. This association is supported by studies reporting a higher prevalence of lipid abnormalities [14], metabolic syndrome [15], cardiovascular diseases [16], and sleep disturbances [17] in individuals with AGA.

While these associations suggest a potential relationship between AGA and systemic conditions, particularly cardiometabolic dysfunctions, the extent to which these factors may serve as independent risk factors for AGA remains unclear. This gap is partly due to the fact that prior studies generally examined these variables separately, hence limiting understanding of their combined contribution when assessed simultaneously within the same individual. Therefore, this study aimed to investigate the hormonal, metabolic, and sleep profiles of adult men with AGA and their potential contribution to its development. Exploring and understanding these links may provide novel perspectives on AGA and contribute to a more integrative management approach.

## Methods

### Studied population

This cross-sectional analysis employed retrospective data from the 4th Edition of the São Paulo Epidemiological Sleep Study (EPISONO 2018), conducted to evaluate sleep patterns within a representative cohort of the population of São Paulo, Brazil. The sampling followed the same 3-stage probabilistic cluster methodology used in the previous edition [18]. The Universidade Federal de São Paulo (UNIFESP) Ethics Committee granted ethical approval (No. 0985/2017; 2.252.970) and informed consent was obtained from all participants. Approval for the secondary use of anonymized data in this analysis was obtained (No. 0387/2024; 6.943.083).

The 4th Edition of EPISONO included 769 participants of both sexes aged 20–80 years old. All participants initially completed sleep and health questionnaires at home, followed by in-person assessments at the Instituto do Sono (São Paulo, Brazil). These assessments were composed of additional sleep questionnaires, anthropometric measurements, and overnight polysomnography (PSG). Fasting blood samples were taken the following morning. For the current study, the inclusion criteria were male participants (n = 317) who answered the AGA-related question in the EPISONO general health questionnaire [18]. Questions about the presence of specific dermatological diseases were assessed, as follows: AGA, acne, psoriasis, dermatitis, and vitiligo. Those who answered affirmatively were classified as having AGA, while those who answered negatively were categorized as non-AGA. Male participants who did not answer the AGA-related question and women individuals of the EPISONO initial sample were excluded from the analysis of the current study.

A subset of these data, including age, body mass index (BMI), metabolic syndrome, and OSA diagnosis, was previously analyzed in a separate study focusing on AGA and iron metabolism in both sexes [19]. In the present analysis, only male participants were included, a different set of covariates was used for the propensity score matching (PSM) procedure, and OSA was categorized into two severity levels. These methodological distinctions resulted in a distinct analytical sample and different outcome patterns.

### Clinical data

Anthropometric measurements, including neck, hip, and waist circumferences, were recorded. Height and weight were measured to calculate BMI. Medical history, medication use, and blood pressure measurements were also collected. Comorbidities were diagnosed according to standardized criteria, as follows: hypertension (blood pressure ≥ 140/90 mmHg or current use of antihypertensive medication) [20]; diabetes (fasting glucose ≥ 126 mg/dL, glycated hemoglobin [HbA1c] ≥ 6.5%, or use of antidiabetic medication) [21]; dyslipidemia (high-density lipoprotein [HDL] cholesterol < 40 mg/dL in men, < 50 mg/dL in women, triglycerides ≥ 150 mg/dL, or use of lipid-modifying therapy) [22]; visceral obesity (waist circumference ≥ 102 cm in men and ≥ 88 cm in women) [23]; and metabolic syndrome (defined by the presence of at least three of the above conditions) [24].

### Biochemical and hormonal assessment

Blood samples were analyzed using standardized techniques at the Division of Diagnostic Medicine of the Associação Fundo de Incentivo à Pesquisa (AFIP). Analysis included glycemic markers (fasting glucose and glycated hemoglobin [HbA1c]), lipid profile (total cholesterol, high-density lipoprotein [HDL] cholesterol, low-density lipoprotein [LDL] cholesterol, very-low-density lipoprotein [VLDL] cholesterol, and triglycerides), cardiac biomarkers (B-type natriuretic peptide [BNP] and N-terminal pro-B-type natriuretic peptide [NT-proBNP]), high-sensitivity C-reactive protein (hs-CRP) as an inflammatory mediator, and sex hormones (luteinizing hormone [LH], follicle-stimulating hormone [FSH], total testosterone, free testosterone, 17-β-estradiol, progesterone, and prolactin). Cardiovascular risk was classified as low to moderate (≤ 0.3 mg/dL) or high (> 0.3 mg/dL) based on hs-CRP levels.

### Sleep assessments

Sleep parameters were assessed using validated questionnaires and PSG. Sleep quality and daytime sleepiness were assessed using the Pittsburgh Sleep Quality Index (PSQI) and the Epworth Sleepiness Scale (ESS). The PSQI is a 19-item self-report questionnaire evaluating sleep quality over the past 30 days, with scores ≥ 5 indicating poor sleep quality [25, 26]. The ESS measures the likelihood of falling asleep in 8 daily situations; scores > 10 indicate excessive daytime sleepiness [27, 28]. The Insomnia Severity Index (ISI) was used to evaluate the nature and severity of insomnia over the past month. The ISI consists of a 5-point Likert scale, with total scores ranging from 0 to 28; a cutoff score of ≥ 15 indicates clinically significant insomnia [29–31].

OSA was diagnosed using type I PSG conducted with the EMBLA® S7000 system (Embla Systems, Inc., Broomfield, CO, USA). The PSG protocol included electroencephalogram, electrooculogram, surface electromyogram, electrocardiogram, airflow, thoracic and abdominal respiratory effort, snoring, body position, oxyhemoglobin saturation, and pulse rate measurements. Data were scored by trained technicians according to the American Academy of Sleep Medicine (AASM) guidelines [32]. OSA was defined as an apnea–hypopnea index (AHI) ≥ 5 events per hour, with severity classified as normal-to-mild (AHI < 15 events/hour) and moderate-to-severe (AHI ≥ 15 events/hour).

### Statistical analysis

Statistical analyses were performed using Jamovi 2.3.26 [33] for descriptive measures, group comparisons, and binomial logistic regression, RStudio 4.3 (R 4.3.0) [34] for PSM and conditional logistic regression, and GraphPad Prism 10.6.1 for graphical visualization. The PSM approach [35] was used to match AGA and non-AGA male participants in a 1:1 ratio, with propensity scores estimated via logistic regression implemented in the MatchIt and cobalt packages. The model incorporated age, BMI, waist-to-hip ratio, diabetes, and metabolic syndrome as baseline covariates, selected based on their well-established associations with AGA and cardiometabolic risk [15, 36]. Matching was performed using a nearest-neighbor algorithm without replacement, applying a caliper width of 0.2 on the logit of the propensity score [37]. Covariate balance was assessed using standardized mean differences (SMD), with SMD < 0.10 considered indicative of adequate balance [38].

Comparisons between AGA and non-AGA groups were conducted before and after PSM. Continuous variables were expressed as mean ± standard deviation (SD), and categorical variables as absolute and relative frequencies. Normality was verified with the Shapiro–Wilk test and homoscedasticity with Levene’s test. Prior to matching, normally distributed continuous variables were compared using independent-sample t-tests, and non-normal variables using the Mann–Whitney U test. Categorical variables were analyzed with chi-square or Fisher’s exact tests, with adjusted residuals used to identify significant deviations in observed frequencies. After matching, paired Student’s t-tests or Wilcoxon signed-rank tests were applied to continuous variables according to their distribution and McNemar’s test was used for categorical variables.

Before matching, univariate binomial logistic regression models were fitted to estimate crude odds ratios (ORs) and 95% confidence intervals (CIs) for age, BMI, triglycerides, and OSA categories, with AGA as the dependent variable. These predictors were subsequently entered into a multivariable model to obtain adjusted odds ratios (AORs). After matching, the associations were re-evaluated, and conditional logistic regression stratified by matched pairs was additionally performed with the *clogit* function from the survival package in R. Multicollinearity was assessed using the variance inflation factor (VIF) and model calibration was examined with the Hosmer–Lemeshow test. Statistical significance was defined as two-sided p < 0.05.

## Results

### Sample description

The initial total sample of EPISONO 4th was composed of 769 individuals of both sexes. In the current analysis only the male sample was enrolled (n = 317). Among these men participants, 307 responded to the AGA-related question (self-reported AGA) through the EPISONO general health questionnaire, which was applied to whole primarily sample of EPISONO 4th Edition. Of these, 64 men responded positively and were included in the AGA group, while 243 responded negatively and were included in the non-AGA group of the present study.

Table 1 outlines the baseline characteristics of both groups before and after PSM. Before PSM, the AGA group was significantly older than the non-AGA group (p = 0.010) and had larger neck (p = 0.017) and waist (p = 0.031) circumferences. The prevalence of diabetes (p = 0.043) and metabolic syndrome (p = 0.012) was significantly higher in the AGA group. Trends toward significance indicating higher values in the AGA group were observed for BMI (p = 0.060), hip circumference (p = 0.067), visceral obesity (p = 0.073), and dyslipidemia (p = 0.072).

**Table 1:** Clinical characteristics of the participants

Variables	Before PSM			After PSM		
	Non-AGA (n = 243)	AGA (n = 64)	p value^a^	Non-AGA (n = 64)	AGA (n = 64)	p value^b^
Age (years)	45.6 ± 14.4	50.6 ± 13.8*	0.010	50.5 ± 15.9	50.6 ± 13.8	0.946
BMI (kg/m^2^)	27.4 ± 4.9	28.6 ± 4.9	0.060	28.7 ± 5.6	28.6 ± 4.9	0.967
Neck circumference (cm)	40.2 ± 3.4	41.2 ± 3.3*	0.017	41.2 ± 3.5	41.2 ± 3.3	0.939
Waist circumference (cm)	98.4 ± 13.6	102.7 ± 13.3*	0.031	102.5 ± 14.4	102.7 ± 13.3	0.672
Hip circumference (cm)	102.2 ± 10.2	105.3 ± 10.7	0.067	104.8 ± 11.3	105.3 ± 10.7	0.747
Waist-to-hip ratio	1.0 ± 0.1	1.0 ± 0.1	0.152	1.0 ± 0.1	1.0 ± 0.1	0.856
Comorbidities, n (%)						
Obesity	57 (23.8)	22 (34.4)	0.085	23 (35.9)	22 (34.4)	1.000
Visceral obesity	84 (34.7)	30 (46.9)	0.073	31 (48.4)	30 (46.9)	1.000
Dyslipidemia	153 (63.0)	48 (75.0)	0.072	44 (68.8)	48 (75.0)	0.540
Hypertension	97 (39.9)	29 (45.3)	0.435	38 (59.4)	29 (45.3)	0.110
Diabetes	38 (15.6)	17 (26.6)^#^	0.043	16 (25.0)	17 (26.6)	1.000
Metabolic syndrome	54 (22.2)	24 (37.5)^#^	0.012	25 (39.1)	24 (37.5)	1.000

Abbrev: AGA, androgenetic alopecia; BMI, body mass index; PSM, propensity score matching

Data are presented as mean ± SD or n (%).

^a^Before PSM: Mann–Whitney U test for continuous variables and chi-square test with adjusted residuals for categorical variables

^b^After PSM: paired t-test or Wilcoxon signed-rank test for continuous variables; McNemar test for categorical variables

^*^Significant when compared to the non-AGA group

^#^Observed frequency is significantly different from the expected frequency.

**Fig. 1:**
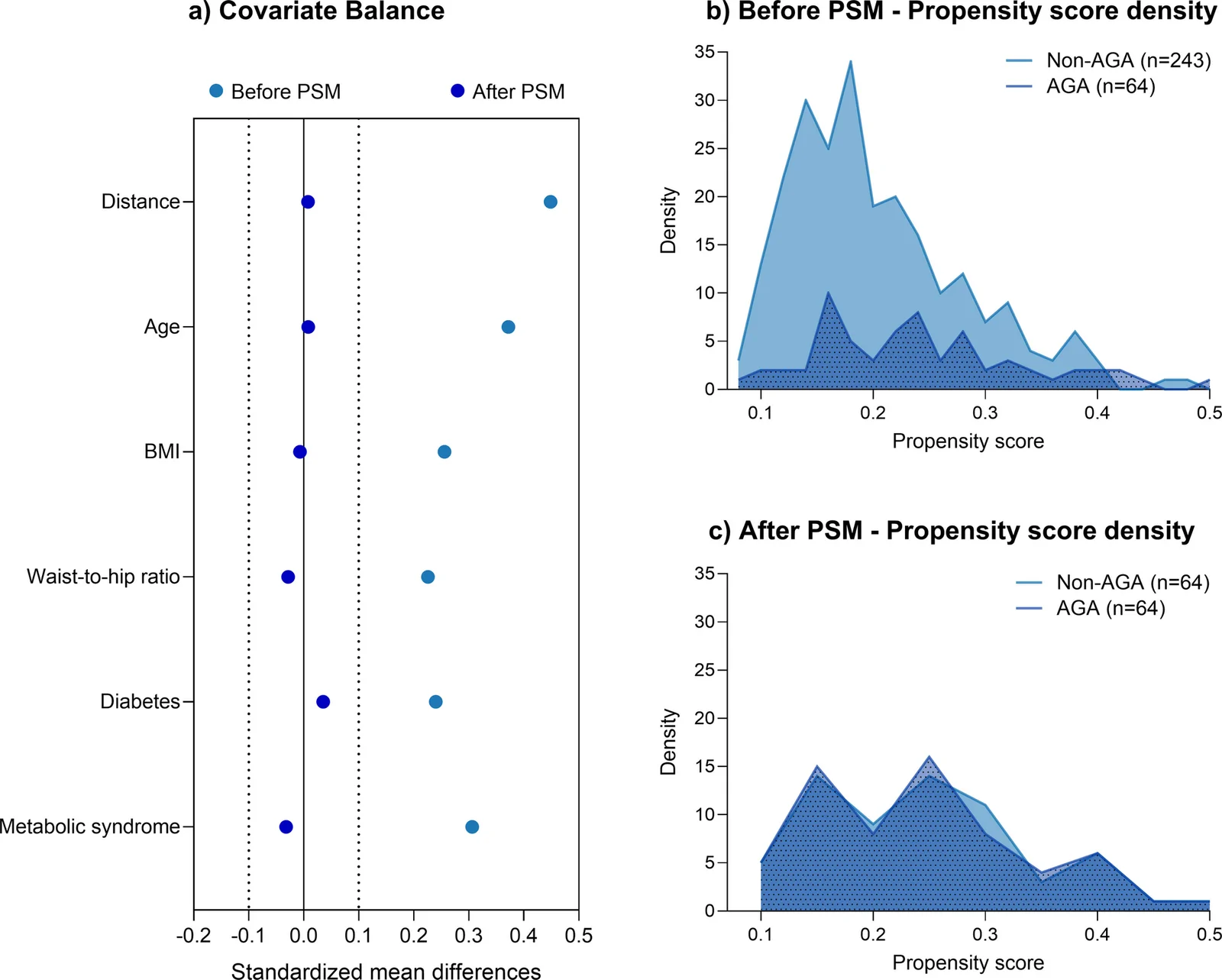
Validation of propensity score matching (PSM). a) Covariate balance plot displaying standardized mean differences (SMD) before and after PSM. Dotted vertical lines indicate an SMD threshold of 0.10, adopted as the criterion for adequate balance, which was achieved for all covariates after matching. b) Propensity score density plot for the AGA (n = 64) and non-AGA (n = 243) groups before matching, illustrating the initial imbalance. c) Propensity score density plot after matching (n = 64 per group), demonstrating improved overlap between groups and confirming the effectiveness of the matching procedure. Propensity scores were estimated using logistic regression including age, body mass index (BMI), waist-to-hip ratio, diabetes, and metabolic syndrome

After PSM, 64 age-matched pairs (AGA and non-AGA groups) were included in the analysis, while 179 non-AGA participants were excluded due to lack of suitable matches within the caliper range. Matching resulted in adequate covariate balance, as indicated by SMD values < 0.10 (Table 2; Fig. 1a). Age (p = 0.946), BMI (p = 0.967), neck (p = 0.939), waist (p = 0.672), and hip circumferences (p = 0.747), as well as waist-to-hip ratio (p = 0.856), no longer differed between groups (Table 1). Metabolic comorbidities were comparable, with no significant differences observed (all p > 0.05). Propensity score density plots demonstrated improved overlap between groups (Fig. 1b-c), confirming the effectiveness of the matching procedure.

**Table 2:** Covariate balance before and after propensity score matching (PSM)

Covariate	Type	SMD	
		Before PSM	After PSM
Propensity score	Distance	0.448	0.008
Age	Continuous	0.372	0.008
BMI	Continuous	0.256	0.007
Waist-to-hip ratio	Continuous	0.226	0.028
Diabetes	Binary	0.240	0.035
Metabolic syndrome	Binary	0.306	0.032

Abbrev: BMI, body mass index; SMD, standardized mean difference; PSM, propensity score matching

SMD < 0.10 indicates adequate covariate balance

### Hormonal and biochemical parameters

Before PSM, hormonal analysis revealed significantly lower total (p = 0.025) and free (p = 0.011) testosterone levels in the AGA group compared to the non-AGA group. The AGA group exhibited significantly lower HDL cholesterol levels (p = 0.041), higher triglyceride concentrations (p = 0.015), and elevated BNP levels (p = 0.004). When hs-CRP levels were stratified by cardiovascular risk, a significantly higher proportion of individuals in the AGA group were classified as high-risk compared to the non-AGA group (42.2% *vs.* 27.7%; p = 0.025). Although mean triglycerides remained higher in the AGA group (211.6 ± 139.9 *vs.* 166.6 ± 83.1), this difference was no longer statistically significant (p = 0.120). Overall, hormonal and biochemical parameters were comparable between groups after PSM (all p > 0.05) (Table 3).

**Table 3:** Biochemical and hormonal parameters

Variables	Before PSM			After PSM		
	Non-AGA (n = 243)	AGA (n = 64)	p value^a^	Non-AGA (n = 64)	AGA (n = 64)	p value^b^
Hormonal parameters						
FSH (mIU/mL)	4.9 ± 4.4	4.8 ± 4.3	0.768	4.9 ± 4.0	4.8 ± 4.3	0.468
LH (mIU/mL)	3.8 ± 2.1	3.6 ± 1.9	0.650	3.8 ± 2.2	3.6 ± 1.9	0.634
Progesterone (ng/mL)	0.2 ± 0.1	0.2 ± 0.1	0.930	0.2 ± 0.1	0.2 ± 0.1	0.281
Total testosterone (ng/dL)	578.2 ± 245.1	520.7 ± 278.7*	0.025	563.8 ± 301.0	520.7 ± 278.7	0.358
Free testosterone (ng/dL)	11.3 ± 3.9	10.3 ± 4.9*	0.011	10.6 ± 4.4	10.3 ± 4.9	0.718
Estradiol (pg/mL)	23.8 ± 9.1	24.8 ± 11.4	0.661	23.4 ± 8.3	24.8 ± 11.4	0.563
Prolactin (ng/mL)	13.6 ± 18.9	10.5 ± 5.2	0.205	14.3 ± 28.6	10.5 ± 5.2	0.703
Biochemical parameters						
Fasting glucose (mg/dL)	106.7 ± 39.6	116.4 ± 53.1	0.191	111.1 ± 42.3	116.4 ± 53.1	0.847
HbA1c (%)	7.7 ± 26.0	6.2 ± 1.4	0.381	12.5 ± 50.4	6.2 ± 1.4	0.834
Total cholesterol (mg/dL)	189.1 ± 44.3	194.8 ± 45.4	0.281	188.3 ± 43.7	194.8 ± 45.4	0.424
HDL cholesterol (mg/dL)	44.5 ± 11.2	42.5 ± 12.6*	0.041	44.6 ± 11.6	42.5 ± 12.6	0.070
LDL cholesterol (mg/dL)	111.2 ± 34.0	112.0 ± 36.7	0.819	111.1 ± 35.1	112.0 ± 36.7	0.890
VLDL cholesterol (mg/dL)	31.1 ± 13.9	35.1 ± 15.1	0.070	32.8 ± 15.0	35.1 ± 15.1	0.403
Triglycerides (mg/dL)	166.9 ± 97.1	211.6 ± 139.9*	0.015	166.6 ± 83.1	211.6 ± 139.9	0.120
BNP (pg/mL)	16.1 ± 26.6	22.1 ± 35.2*	0.004	19.5 ± 32.7	22.3 ± 35.4	0.562
NT‐proBNP (pg/mL)	66.1 ± 178.3	147.0 ± 602.5	0.182	81.3 ± 115.1	147.0 ± 602.5	0.368
hs-CRP (mg/dL)	0.4 ± 0.8	0.5 ± 0.7	0.078	0.5 ± 0.8	0.5 ± 0.7	0.339
CDV risk based on hs-CRP levels, n (%)			0.025			0.216
*Low-to-moderate risk (*≤ *0.3 mg/dL)*	175 (72.3)	37 (57.8)		45 (70.3)	37 (57.8)	
*High risk (*> *0.3 mg/dL)*	67 (27.7)	27 (42.2)^#^		19 (29.7)	27 (42.2)	

Abbrev: AGA, androgenetic alopecia; BNP, B-type natriuretic peptide; CDV, cardiovascular; HbA1c, glycated hemoglobin; FSH, follicle-stimulating hormone; HDL, high-density lipoprotein; hs-CRP, high-sensitivity C-reactive protein; LDL, low-density lipoprotein; LH, luteinizing hormone; NT-proBNP, N-terminal pro-B-type natriuretic peptide; PSM, propensity score matching; VLDL, very-low-density lipoprotein

Data are presented as mean ± SD or n (%).

^a^Before PSM: Mann–Whitney U test for continuous variables and chi-square test with adjusted residuals for categorical variables

^b^After PSM: paired t-test or Wilcoxon signed-rank test for continuous variables; McNemar test for categorical variables

^*^Significant when compared to the non-AGA group

^#^Observed frequency is significantly different from the expected frequency.

### Sleep measures

No significant differences were found in PSQI, ESS, or ISI scores between groups before or after PSM. Moderate-to-severe OSA was more prevalent in the AGA group before matching (37.5% vs. 24.7%; p = 0.041), but this difference disappeared after PSM (p = 1.000) (Table 4).

**Table 4:** Sleep parameters of androgenetic alopecia (AGA) and non-AGA groups before and after propensity score matching (PSM)

Variables	Before PSM			After PSM		
	Non-AGA (n = 243)	AGA (n = 64)	p value^a^	Non-AGA (n = 64)	AGA (n = 64)	p value^b^
PSQI score, mean ± SD^†^	10.5 ± 3.1	10.1 ± 2.4	0.641	10.7 ± 3.4	10.3 ± 2.5	0.503
PSQI ≥ 5, n (%)	216 (96.9)	62 (100.0)	0.353	53 (94.6)	62 (100.0)	0.248
PSQI components, mean ± SD						
Sleep quality	2.1 ± 1.1	2.2 ± 1.0	0.817	2.2 ± 1.1	2.2 ± 1.0	0.988
Sleep latency	1.4 ± 1.1	1.1 ± 1.1	0.122	1.6 ± 1.2	1.2 ± 1.1	0.058
Sleep duration	1.2 ± 0.8	1.1 ± 0.8	0.301	1.1 ± 0.9	1.0 ± 0.8	0.811
Sleep efficiency	0.6 ± 0.9	0.5 ± 0.9	0.828	0.6 ± 1.0	0.5 ± 0.9	0.611
Sleep disturbance	1.5 ± 0.6	1.4 ± 0.5	0.839	1.5 ± 0.6	1.4 ± 0.5	0.278
Medication use	2.4 ± 0.9	2.4 ± 0.8	0.629	2.4 ± 0.8	2.4 ± 0.8	0.715
Daytime dysfunction	1.5 ± 0.7	1.5 ± 0.8	0.958	1.5 ± 0.7	1.5 ± 0.8	0.985
ESS score, mean ± SD	8.3 ± 4.6	8.7 ± 5.1	0.840	7.9 ± 4.8	8.7 ± 5.1	0.378
ESS > 10, n (%)	72 (29.6)	22 (34.4)	0.464	20 (31.3)	22 (34.4)	0.845
ISI score, mean ± SD	7.2 ± 6.4	6.3 ± 5.4	0.548	8.0 ± 6.3	6.3 ± 5.4	0.130
OSA categories, n (%)			0.041			1.000
Normal-to-mild (AHI < 15/hour)	183 (75.3)	40 (62.5)		40 (62.5)	40 (62.5)	
Moderate-to-severe (AHI ≥ 15/hour)	60 (24.7)	24 (37.5)^#^		24 (37.5)	24 (37.5)	

Abbreviations: AGA, androgenetic alopecia; AHI, apnea–hypopnea index; ESS, Epworth Sleepiness Scale; ISI, Insomnia Severity Index; OSA, obstructive sleep apnea; PSM, propensity score matching; PSQI, Pittsburgh Sleep Quality Index

Data are presented as mean ± SD or n (%).

^a^Before PSM: Mann–Whitney U test for continuous variables and chi-square test with adjusted residuals for categorical variables

^b^After PSM: paired t-test or Wilcoxon signed-rank test for continuous variables; McNemar test for categorical variables

^*^Significant when compared to the non-AGA group

^#^Observed frequency is significantly different from the expected frequency.

^†^1Missing data were identified for PSQI scores in both AGA and non-AGA groups before and after PSM.

### Factors influencing the risk of AGA

In the univariate logistic regression analyses before PSM, age (OR = 1.025, 95%CI = 1.005–1.045; p = 0.014) and higher triglycerides (OR = 1.003, 95%CI = 1.001–1.006; p = 0.005) were significantly associated with increased odds of AGA, with each additional year of age and each 1 mg/dL rise in triglycerides increasing the odds by 2.5% and 0.3%, respectively. Moderate-to-severe OSA was associated with a 1.83-fold increase in the odds of being in the AGA group (OR = 1.830, 95%CI = 1.020–3.282; p = 0.043), whereas BMI showed no significant association. In the multivariable model, age (AOR = 1.023, 95%CI = 1.002–1.045; p = 0.037) and triglycerides (AOR = 1.003, 95%CI = 1.000–1.005; p = 0.018) remained significant predictors, whereas the association with moderate-to-severe OSA was no longer significant (AOR = 1.337, 95%CI = 0.691–2.589; p = 0.389) (Table 5).

**Table 5:** Logistic regression analyses of factors associated with androgenetic alopecia before and after propensity score matching

Variables	Univariate analysis		Multivariable analysis	
	OR (95% CI)	p-value	AOR (95% CI)	p-value
Before PSM^a^				
Age (years)	1.025 (1.005–1.045)	0.014	1.023 (1.002–1.045)	0.037
BMI (kg/m^2^)	1.051 (0.995–1.110)	0.073	1.036 (0.974–1.101)	0.259
Triglycerides (mg/dL)	1.003 (1.001–1.006)	0.005	1.003 (1.000–1.005)	0.018
OSA categories (moderate-to-severe *vs.* normal-to-mild)	1.830 (1.020–3.282)	0.043	1.337 (0.691–2.589)	0.389
After PSM^b^				
Age (years)	1.001 (0.963–1.041)	0.945	1.011 (0.951–1.075)	0.718
BMI (kg/m^2^)	0.998 (0.925–1.078)	0.966	1.033 (0.908–1.176)	0.621
Triglycerides (mg/dL)	1.004 (1.000–1.008)	0.043	1.004 (1.000–1.009)	0.039
OSA categories (moderate-to-severe *vs.* normal-to-mild)	1.000 (0.464–2.157)	1.000	1.131 (0.489–2.619)	0.773

Abbrev: AGA, androgenetic alopecia; AOR, adjusted odds ratio; BMI, body mass index; CI, confidence interval; OR, odds ratio; OSA, obstructive sleep apnea; PSM, propensity score matching

^a^Before PSM: binary logistic regression

^b^After PSM: conditional logistic regression

After PSM, conditional logistic regression indicated that triglycerides remained the only variable associated with AGA in both univariate (OR = 1.004, 95%CI = 1.000–1.008; p = 0.043) and multivariable analyses (AOR = 1.004, 95% CI = 1.000–1.009; p = 0.039), whereas age, BMI, and OSA categories were not statistically associated with AGA (Table 5).

## Discussion

This study assessed metabolic, hormonal, and sleep-related parameters in adult men with AGA. The main finding was the consistent involvement of serum triglycerides as a predictor of AGA, after balancing groups for age and key metabolic factors. This result suggests a potential contribution of lipid metabolism, particularly hypertriglyceridemia, to follicle impairment. No significant differences emerged in subjective sleep aspects. In respect of objective sleep measures, although moderate-to-severe OSA was more common in the AGA group and initially associated with higher risk, this association lost significance after adjustment, indicating possible confounding by age or related metabolic conditions.

These findings are consistent with previous evidence linking AGA to disturbances in lipid metabolism. A meta-analysis of 19 observational studies confirmed this pattern, showing significantly elevated serum levels of total cholesterol, triglycerides, and LDL, and reduced HDL levels in individuals with AGA compared to controls [14]. Similarly, another study [39] observed higher levels of triglycerides in an AGA sample, which was suggested as a high-specificity biomarker in this hair condition. A recent study identified a significant association between AGA severity and lipid parameters; as AGA severity increased, total cholesterol, HDL, LDL, and the cholesterol/HDL ratio showed progressively abnormal values, although the association with triglycerides was not statistically significant [40]. The present study provides novel evidence that elevated serum triglyceride levels may represent a potential predictor of AGA. The association was evident in the analyses performed before matching and persisted after balancing groups for age, BMI, waist-to-hip ratio, diabetes, and metabolic syndrome, indicating that triglycerides may play an independent role in AGA pathophysiology. Despite these specific associations already reported in the literature, the underlying mechanisms linking dyslipidemia to AGA pathogenesis remain poorly understood.

It is well established that lipid metabolism plays a critical role in hair follicle (HF) physiology and in the regulation of hair growth. Circulatory lipoproteins, particularly LDL, act as cholesterol carriers to the HF. As a result, systemic lipid imbalances may disrupt HF function [41], potentially interfering with the hair cycle by shortening the anagen phase, a hallmark of AGA [5]. Findings further highlighted the relevance of lipid metabolism in HF biology, demonstrating reduced lipid activity in the scalp of individuals with AGA [42]. Lipid supplementation stimulated dermal papilla cell proliferation and upregulated trichogenic gene expression via activation of hypoxia-inducible factor 1-alpha (HIF-1α), a key transcription factor involved in cellular responses to low oxygen levels. This treatment promoted hair shaft elongation in HF organoids, indicating a direct HIF-1-mediated effect on hair growth.

As previously noted, dyslipidemia should be considered a cardiovascular risk factor in individuals with AGA, particularly in those with early-onset [16, 43]. Supporting this view, a meta-analysis of 31 studies involving more than 29,000 individuals with alopecia, mostly cases of AGA, demonstrated an increased risk of coronary heart disease, insulin resistance, hyperinsulinemia, metabolic syndrome, and elevated serum levels of total cholesterol and triglycerides [44]. In our study, individuals with AGA exhibited a higher cardiovascular risk based on hs-CRP levels, as well as increased rates of diabetes and metabolic syndrome compared to the non-AGA group. Although these associations were not maintained after PSM, likely due to confounding by age and metabolic variables, numerical differences persisted. These findings underscore the importance of monitoring both inflammatory and lipid-related markers in individuals with AGA and warrant further investigation, particularly given the occurrence of dyslipidemia in isolation, without the presence of other comorbidities, within the AGA context.

Before PSM, the average age of the AGA group was significantly higher than that observed in the non-AGA group (50.6 *vs.* 45.6, respectively). This result was expected, as the progression of AGA and its pattern distribution increase with age, although the first signs of this condition, such as hair thinning, can begin in early adulthood [5, 45]. Some studies have found lower mean ages in the AGA samples in different populations, such as 26 [40], 36 [46] and 41 years old [47], which may reflect differences across different local regions. However, AGA generally affects 30 to 50% of male individuals by the age of 50 years old [3]. The presence of clinical comorbidities is also anticipated, as its incidence increases with age.

Regarding the hormonal profile in AGA, although elevated levels of dihydrotestosterone (DHT) are well-established, findings on total and free testosterone remain inconsistent, and it can vary depending on the age and hormonal status [48]. Men with early-onset AGA (< 35 years old) may exhibit a hormonal profile similar to that found in women with polycystic ovary syndrome, characterized by decreased follicle-stimulating hormone (FSH), and increased luteinizing hormone (LH), total testosterone, androstenedione, and 17α-hydroxyprogesterone (17αOH-P), and low SHBG [49, 50]. These men also tend to show adverse cardiometabolic outcomes, such as hypertension, insulin resistance and higher BMI. Conversely, in cases where hypogonadism is present, total and free testosterone levels may be reduced, reflecting impaired gonadal function [49]. In our sample, lower testosterone levels were observed in the AGA group, suggesting that a distinct gonadal profile may exist between men with early-onset and those with late-onset AGA, in line with the mean age of our sample. The endocrine profile in individuals with AGA can vary depending on the associated comorbidities, which may indicate distinct clinical scenarios. It should be noted that testosterone can be reduced in cases of OSA, as stated in previous studies [51, 52], including by our group [51]. This calls attention to the possible influence of sleep disorders in the reduction of testosterone levels in AGA, particularly OSA.

In relation to sleep, a study [17] reported a significant association between severe AGA and shorter sleep duration, poorer sleep quality, and increased risk for OSA, as indicated by higher STOP-BANG scores. In our study, the prevalence of moderate-to-severe OSA was initially higher in the AGA group, but this association lost significance after PSM, indicating a confounding effect of age and related metabolic factors [53]. No significant differences were observed in subjective sleep assessments (PSQI, ESS, and ISI) between groups, which may reflect the limited sensitivity of these tools in capturing sleep alterations specifically associated with AGA. Although the relationship between AGA and OSA remains incompletely understood, metabolic alterations, such as dyslipidemia and hormonal imbalances may contribute to this association. Hypoxia-induced dyslipidemia, commonly observed in OSA, has been linked to worsening metabolic profiles and may also influence AGA progression [54]. These findings underscore the need for longitudinal studies to clarify the interplay between AGA, sleep disorders, and metabolic dysfunction. Clinically, our results support routine metabolic screening, particularly lipid profile assessment, among patients with AGA. Early identification and management of dyslipidemia may not only provide cardiovascular benefits but also potentially influence the clinical progression of AGA.

### Strengths and limitations

The main strengths of this study include the use of PSG, the gold standard exam for diagnosing OSA, ensuring high diagnostic accuracy. The study was conducted in a large and demographically diverse sample from São Paulo, the largest metropolis in the Southern hemisphere, which enhances the external validity of the findings. The analysis of sleep quality in individuals with AGA addresses a rarely explored topic in the medical literature, contributing to a better understanding of potential links between AGA, lipid metabolism, and sleep disturbances, especially OSA. This triple interaction, which has been only sparsely examined in previous studies, represents the main contribution of our work by highlighting a novel association between lipid metabolism, hormonal profiles, and OSA in individuals with AGA.

Some limitations must be acknowledged. First, The EPISONO study was not primarily intended to evaluate AGA, and its classification relied on self-reported data without clinical verification, which may have introduced recall bias. Specific details regarding AGA onset, symptoms, and severity were not available, precluding a more in-depth assessment of its clinical features. A self-reported clinical questionnaire including some of specific dermatological issues could be selected by the participants, which may have introduced recall bias and led to variability and misclassification. However, the fact that individuals selected a specific type of skin disorders means that they know their skin conditions. According to Short et al. [55], self-reported conditions are a relevant and accurate method of clinical examination and estimative of frequency of diseases, which should be valued in clinical assessments. Second, the original sample size was relatively small and unevenly distributed (AGA: n = 64; non-AGA: n = 243), which may have influenced statistical power and group comparability. Because AGA status was self-reported within a population-based epidemiological study, the number of individuals with AGA reflects the natural distribution of this condition in the source population rather than an investigator-defined sample size. This sample size may reflect an approximate frequency of AGA and consistency with previously described epidemiological estimates. Given that the prevalence of AGA can vary between 30 and 80%, depending on the age range, our sample size can indicate a plausible and reliable frequency of this hair disorder. To mitigate these effects, PSM was applied to balance the groups using age, body mass index, waist-to-hip ratio, diabetes, and metabolic syndrome as baseline covariates, resulting in matched pairs with equal sample sizes (AGA: n = 64; non-AGA: n = 64). PSM has inherent limitations, as it excludes unmatched individuals, reducing the final sample size and potentially limiting generalizability, while also accounting only for measured variables, which leaves room for residual confounding due to unmeasured factors.

## Conclusions

This study reinforces the association between AGA and lipid metabolism, identifying elevated serum triglyceride levels as a potential independent marker of AGA. Although no significant differences were observed in subjective sleep quality and hormonal alterations appeared to vary with age, the initial association between AGA and moderate-to-severe OSA, despite losing significance in the PSM analysis, suggests that there is a complex interplay between AGA and age-related and metabolic factors.

Our findings indicate that routine metabolic screening, including lipid profile assessment, should be considered in men with AGA to enable early identification of cardiometabolic risk. Longitudinal studies are warranted to elucidate causal pathways and to determine whether early metabolic intervention may influence the clinical progression of AGA and its related comorbidities.

## Data Availability

The data that support the findings of this study are available from the corresponding author upon reasonable request.
